# NKX2-1 re-expression induces cell death through apoptosis and necrosis in dedifferentiated thyroid carcinoma cells

**DOI:** 10.1371/journal.pone.0259558

**Published:** 2021-11-08

**Authors:** Yuko Ito, Fumihiko Furuya, Katsumi Taki, Hideaki Suzuki, Hiroki Shimura

**Affiliations:** 1 Department of Laboratory Medicine, School of Medicine, Fukushima Medical University, Fukushima, Fukushima, Japan; 2 Third Department of Internal Medicine, Interdisciplinary Graduate School of Medicine and Engineering, University of Yamanashi, Chuo, Yamanashi, Japan; 3 Department of Internal Medicine, Fujiyoshida Municipal Medical Center, Fujiyoshida, Yamanashi, Japan; 4 Department of Clinical Laboratory Sciences, School of Health Sciences, Fukushima Medical University, Fukushima, Fukushima, Japan; Columbia University, UNITED STATES

## Abstract

NK2 homeobox 1 (NKX2-1) is a thyroid transcription factor essential for proper thyroid formation and maintaining its physiological function. In thyroid cancer, NKX2-1 expression decreases in parallel with declined differentiation. However, the molecular pathways and mechanisms connecting NKX2-1 to thyroid cancer phenotypes are largely unknown. This study aimed to examine the effects of NKX2-1 re-expression on dedifferentiated thyroid cancer cell death and explore the underlying mechanisms. A human papillary thyroid carcinoma cell line lacking NKX2-1 expression was infected with an adenoviral vector containing *Nkx2-1*. Cell viability decreased after *Nkx2-1* transduction and apoptosis and necrosis were detected. Arginase 2 (*ARG2*), regulator of G protein signaling 4 (*RGS4*), and *RGS5* mRNA expression was greatly increased in *Nkx2-1*-transducted cells. After suppressing these genes by siRNA, cell death, apoptosis, and necrosis decreased in RGS4 knockdown cells. These findings demonstrated that cell death was induced via apoptosis and necrosis by NKX2-1 re-expression and involves RGS4.

## Introduction

Thyroid transcription factors (TTFs), namely NK2 homeobox 1 (NKX2-1, also known as TTF-1), forkhead box E1 (FOXE1), paired box 8 (PAX8), and hematopoietically expressed homeobox (HHEX), are fundamental for proper thyroid gland formation and maintaining the functional differentiated state of the adult thyroid [1]. NKX2-1, FOXE1, and PAX8 bind to the DNA and regulate thyroid-specific genes that drive thyroid hormone synthesis, such as thyroglobulin (*TG*), thyroid peroxidase (*TPO*), thyroid stimulating hormone receptor (*TSHR*), and sodium iodide symporter (*NIS*, also known as solute carrier family 5 member 5). Simultaneous TTF expression is unique to the thyroid follicular cells, although each TTF is expressed in other tissues in adults. NKX2-1 is expressed in the lung and nervous system, and FOXE1 and PAX8 are expressed in several tissues [1].

Since both cell proliferation and differentiation are involved in normal and cancer development processes, the genes critical for development act on tumorigenesis in various tissues where they are expressed, including the thyroid [2]. Several reports have described the association between NKX2-1 expression and carcinogenesis in non-thyroid tissues, as well as in thyroid glands. NKX2-1 is required for physiological lung function maintenance in addition to its developmental roles. In lung adenocarcinoma, NKX2-1 expression is increased [3] and confers a good prognosis [4]. Kusakabe *et al*. have reported that conditional *Nkx2-1* knockout in mice produces a disorganized thyroid with degenerating follicular cells [5]. Consequently, their thyroid cell proliferation rate is approximately 2-fold higher than that in wild-type mice, which contributes to a high incidence of genotoxic carcinogen-induced thyroid tumors [6].

A multistep carcinogenesis model for dedifferentiation in thyroid cancer has been proposed based on general concepts and specific pathways [7]. Genomic instabilities induced by risk factors result in early genetic alterations involving the mitogen-activated protein kinase (MAPK) signaling pathway. Oncogenic MAPK signaling activation leads to later genetic alterations, such as *TP53* and *CTNNB1* mutations, which involve other signaling pathways, cell cycle regulators, and various adhesion molecules. Accelerating the interactions between genomic instability and genetic alterations promotes the progression from well-differentiated to undifferentiated thyroid carcinoma [7]. Immunohistochemical analysis showed that NKX2-1 expression decreases in thyroid cancer, corresponding to the progressive thyroid tumor dedifferentiation [8]. Dedifferentiated thyroid cancers are commonly defined as differentiated or poorly differentiated thyroid cancers, which, during tumor progression, lose their ability to uptake and concentrate radioiodine and produce thyroglobulin in some cases [9]. Ros *et al*. have reported that the expression of thyroid-specific genes, such as *TG*, *TPO*, and *TSHR*, is decreased or lost in dedifferentiated thyroid cancers with no *NKX2-1* and *PAX8* expression [10]. We have previously reported that both TG and TPO mRNA and protein are re-expressed in dedifferentiated thyroid cancer cells (NKX2-1^−^/PAX8^+^) infected with adenoviral vectors containing *Nkx2-1* (AdNKX2-1), which induces radioiodide organification and retention [11].

Yamaguchi *et al*. have described that *NKX2-1* plays a role as a lineage-survival oncogene in lung adenocarcinomas; however, it also inhibits invasion, metastasis, and progression, paradoxically conferring a good prognosis [12]. Similarly, while we demonstrated the TG and TPO re-expression in dedifferentiated thyroid cancer cells by *Nkx2-1* transduction [11], we coincidentally observed cell death. The observations in the NKX2-1-re-expressed thyroid cancer cells suggested that NKX2-1 suppresses thyroid cancer cell progression, as well as maintains the functions of normal thyroid follicular cells. However, the molecular pathways and mechanisms connecting TTFs to thyroid dysgenesis and thyroid cancer are largely unknown [1]. Therefore, this study aimed to examine the effects of NKX2-1 re-expression on cell death in dedifferentiated thyroid cancer cells lacking NKX2-1 expression. In addition, we analyzed the genes involved in NKX2-1-induced cell death to explore the underlying mechanisms. Based on the GeneChip results, we focused on arginase 2 (*ARG2*), regulator of G protein signaling 4 (*RGS4*), and *RGS5*. ARG2 converts arginine to ornithine and urea. Arginine is involved in numerous cellular metabolic and signaling pathways and also a substrate for nitric oxide synthase. RGS limits the lifetime of signaling events in G protein. RGS4 responds to cardiovascular stress and modulates neuronal signaling, whereas RGS5 controls vascular remodeling.

## Materials and methods

### Cell culture

BHP18-21v and BHP7-13 cells, expressing *PAX8* but not *NKX2-1*, *FOXE1*, *TG*, or *TPO* genes [13], were kindly provided by Prof. J. M. Hershman (University of California; Los Angeles, CA, USA). These cells were derived from primary tumors with histological features of human papillary thyroid carcinoma [14] and maintained in RPMI-1640 containing 10% fetal bovine serum (FBS) in 5% CO_2_ at 37°C. The DNA BHP18-21v cells has already been profiled previously [15]. MDCK (RIKEN RCB0995; Wako, Japan), a canine kidney epithelial cell line expressing PAX8 but not NKX2-1, was cultured in Dulbecco’s modified Eagle medium (DMEM) supplemented with 10% FBS. HepG2, a human hepatocellular cancer cell line that does not express NKX2-1 or PAX8, was purchased from the American Type Culture Collection (Manassas, VA, USA) and cultured in DMEM containing 10% FBS.

### Recombinant adenoviral vector construction

AdNKX2-1 is a ΔE1–ΔE3 recombinant adenovirus expressing rat *Nkx2-1* under the control of the cytomegalovirus (CMV) immediate early promoter. Rat NKX2-1 has 92.4 and >98% homology to the cDNA and amino acid sequence, respectively, of human NKX2-1. We constructed AdNKX2-1 using rat *Nkx2-1* to distinguish between exogenous and endogenous genes. AdNKX2-1 virus construction has been previously described as ’AdTTF-1’ [13]. AdLacZ, which contains the CMV promoter-controlled *lac*Z gene, was purchased from Quantum Biotechnologies (Montréal, QC, Canada) and used as a control. Recombinant adenoviruses were plaque-purified, harvested 72 h after infecting 293 cells, and purified using the ViraTrap^TM^ Adenovirus Purification Maxi Kit (Biomiga; San Diego, CA, USA). The viral titers were determined by tissue culture infectious dose 50 (TCID_50_) using cultured 293 cells.

### Cell viability quantification

BHP18-21v cells (1.2×10^3^ or 3.6×10^3^ cells/well) were seeded in a 96-well culture plate for analyzing time or multiplicity of infection (MOI) dependencies, respectively. The adenoviral vectors were infected at the indicated MOI 24 h after plating BHP18-21v cells and cell viability was quantified at the indicated time after infection using the Cell Counting Kit-8 (CCK-8; Dojindo; Mashiki, Japan) according to the manufacturer’s protocol. After adding 10 μL CCK-8 solution to each well, the plate was incubated for 90 min before measuring the absorbance using a microplate reader (Varioskan LUX; Thermo Fisher Scientific; Waltham, MA, USA).

### Cellular apoptosis and necrosis detection

#### Acridine orange and ethidium bromide staining

BHP18-21v cells cultured in six-well plates were infected with 300 MOI AdNKX2-1 or AdLacZ for 48 h and exposed to 2 μg/mL acridine orange and 2 μg/mL ethidium bromide in phosphate-buffered saline (PBS). The fluorescence was observed under a fluorescence microscope.

#### Hoechst 33342 staining

BHP18-21v cells cultured on cover slips were infected with 300 MOI AdNKX2-1 or AdLacZ for 48 h, fixed with 1% glutaraldehyde, stained by incubating with 2 mM Hoechst 33342 in dark for 30 min at room temperature, and then washed thrice with PBS. The fluorescence was observed under a fluorescence microscope.

#### Realtime assay using annexin V

Annexin V and cell permeability were measured using RealTime-Glo^TM^ Annexin V Apoptosis and Necrosis Assay (Promega; Madison, WI, USA) in accordance with the manufacturer’s recommendations. Briefly, BHP18-21v cells were cultured in a 96-well Nunc^TM^ MicroWell^TM^ Optical-Bottom Plate with Polymer Base (Thermo Fisher Scientific). The cells were infected with 300 MOI AdNKX2-1 or AdLacZ 24 h after seeding for another 24–48 h. Then, the detection reagent containing Annexin V NanoBiT^®^ substrate, CaCl_2_, necrosis detection reagent, Annexin V-SmBiT, and Annexin V-LgBiT, was added to each well of the 96-well plate. The luminescence for apoptosis and fluorescence (485 nm_Ex_/525 nm_Em_) for necrosis were measured at the indicated time after infection using a microplate reader (Varioskan LUX).

#### Caspase activities

Caspase3/7 activities were analyzed using the CellEvent^TM^ Caspase-3/7 Green Detection Reagent (Thermo Fisher Scientific), according to the manufacturer’s instructions. BHP18-21v cells were cultured in 96-well plates, infected with 300 MOI AdNKX2-1 or AdLacZ for 72 h and incubated for 30 min with CellEvent^TM^ Caspase-3/7 Green Detection Reagent (final concentration, 5 μM). The fluorescence was measured at 502 nm_Ex_/530 nm_Em_ using a Varioskan LUX microplate reader.

### Comprehensive gene expression analysis

BHP18-21v cells incubated in 10-cm dishes were infected with 100 MOI AdNKX2-1 or AdLacZ for 6, 12, and 24 h and collected. Total RNA was isolated from each frozen sample using the RNeasy^®^ Mini Kit (QIAGEN; Hilden, Germany) for gene expression analysis. Gene expression profiles were determined using GeneChip^TM^ Human Genome U113 plus 2.0 array (Affymetrix; Santa Clara, CA, USA) according to the manufacturer’s recommendations. Briefly, double-stranded cDNA was synthesized from 8 μg total RNA with oligo d(T)24 T7 primer and transcribed into biotinylated cRNA using the IVT Labeling Kit (Affymetrix). Then, 20 μg biotinylated cRNA was fragmented at 94°C for 35 min and hybridized to the GeneChip array containing >54,000 probe sets. The cRNA probes hybridized to oligonucleotide arrays were stained with streptavidin R-phycoerythrin and scanned using a GeneChip Scanner 3000 (Affymetrix). The scanned data were processed for signal values using Microarray Suite 5.0 software (Affymetrix). All data used for the subsequent analysis passed the quality control criteria, according to the manufacturer’s protocol.

### mRNA level determination

Total RNA was isolated from cultured cells using the RNeasy^®^ Mini Kit. After spectrophotometric quantification, 5 μg total RNA was reverse-transcribed into cDNA using the SuperScript^®^ III First-Strand Synthesis System for RT-PCR (Thermo Fisher Scientific). Oligonucleotide primers, including TaqMan probes for *ARG2* (Hs00982833_m1), *RGS4* (Hs01111690_g1), and *RGS5* (Hs01591223_s1), were purchased from Thermo Fisher Scientific. To normalize the differences in cDNA amount added to the reactions, 18S rRNA or glyceraldehyde-3-phosphate dehydrogenase (*GAPDH*) was amplified as an endogenous control. Primers and probes for 18S rRNA (Hs99999901_s1) and *GAPDH* (Hs99999905_m1) were purchased from Thermo Fisher Scientific. Real-time PCR was performed in 96 sample tubes using the cDNA equivalent to 100 ng RNA with 1× TaqMan^®^ Universal Master Mix II in Uracil-N-Glycosylase (UNG; Thermo Fisher Scientific), 900 nM each primer, and 400 nM TaqMan probe on a StepOnePlus^TM^ Real-Time PCR System (Thermo Fisher Scientific). The cycling conditions were: 2 min initial phase at 50°C, followed by 10 min at 95°C for optimal UNG enzyme activity, and 50 cycles of 15 s at 95°C and 1 min at 60°C.

Gene expression data were analyzed using the comparative C_T_ method (ΔΔC_T_). 18S rRNA or *GAPDH* was used as the reference gene. Missing values occurred when gene expression was below the detection limit after 45 cycles.

### Western blot analysis

Cell lysates were prepared using EzApply cell lysis buffer (ATTO Corporation; Tokyo, Japan), as described by the manufacturer. The protein concentrations were determined using the Bradford method with bovine serum albumin as the standard (Takara Bio Inc.; Kusastu, Japan). To detect NKX2-1, ARG2, RGS4, and RGS5, whole cell lysates (10 or 30 μg each) were electrophoretically resolved on a 12.5% sodium dodecyl sulfate polyacrylamide gel, and the proteins were transferred to polyvinylidene difluoride membranes using QBlot kits (ATTO Corp). The membranes were incubated with the blocking buffer, EzBlock Chemi (ATTO Corp), and incubated with the following primary antibodies: rabbit monoclonal anti-TTF1 (1:2000 dilution; ab76013; Abcam; Cambridge, UK), rabbit monoclonal anti-Arg2 (1:1000, ab137069, Abcam), rabbit monoclonal anti-RGS4 (1:1000; 15129S; Cell Signaling Technology; Danvers, MA, USA), rabbit polyclonal anti-RGS5 (1:500, ab196799, Abcam), and mouse monoclonal anti-RGS5 (1:100; sc-390245; Santa Cruz Biotechnology; Dallas, TX, USA). Incubation with anti-RGS4 antibody was carried out at 4°C overnight, and those with the other antibodies were performed at room temperature for 60 min. The membranes were washed with Tris-buffered saline with 0.1% Tween 20 and then incubated with secondary antibodies: goat anti-rabbit IgG H&L (HRP) (ab205718, Abcam) or Amersham ECL anti-mouse IgG, Horseradish Peroxidase-Linked Species-Specific Whole Antibody (NA931; GE Healthcare; Chicago, IL, USA), and signals were detected using EzWestLumi plus (ATTO Corp). To detect protein loading, the blots were stripped and incubated with anti-GAPDH antibody (sc-520906, Santa Cruz Biotechnology). The relative band intensities were quantified using an image analysis software (ImageQuant TL 8.1, GE Healthcare).

### siRNA transfection to target ARG2 and RGS4

Three predesigned validated siRNAs for human *ARG2* (*Silencer*^®^ Select; s1571, s1572, and s1573 [siARG2#1, siARG2#2, and siARG2#3, respectively]) and *RGS4* (*Silencer*^®^ Select; s11992, s230181, and s230182 [siRGS4#1, siRGS4#2, and siRGS4#3, respectively]) were purchased from Thermo Fisher Scientific, because silencing efficiency considerably varies among siRNAs.

BHP18-21v cells were transfected with 5 nM each siRNA using Lipofectamine^TM^ RNAiMAX Transfection Reagent (Thermo Fisher Scientific). siRNA transfection was performed according to the manufacturer’s protocol for the “reverse transfections.” First, each siRNA was diluted using Opti-MEM^®^ medium (Thermo Fisher Scientific). Lipofectamine RNAiMAX was then added to the diluted siRNA. After incubating the complexes for 15 min at room temperature, the cells diluted in growth medium were added, which were infected with adenoviral vectors 24 h after siRNA transfection, further incubated for the indicated times, and assayed. To monitor siRNA delivery efficiency, we measured *GAPDH* (*Silencer*^®^ Select GAPDH Positive Control; Thermo Fisher Scientific) mRNA levels using real-time PCR. Nonspecific siRNA (*Silencer*^®^ Select Negative Control No.1 [siNegative]; Thermo Fisher Scientific) was used as a negative control.

### Statistical analysis

Statistical analyses were performed using the statistical software IBM SPSS Statistics 26 (IBM; Armonk, NY, USA). All data are expressed as mean ± standard error of the mean (SEM). Differences in variables between AdLacZ- and AdNKX2-1-infected cells were assessed using Student’s *t*-test, and those among the siRNA groups were analyzed using one-way analysis of variance (ANOVA). Statistical significance was set at *p*<0.05.

## Results

### AdNKX2-1-induced BHP18-21v cell death

**Fig 1:**
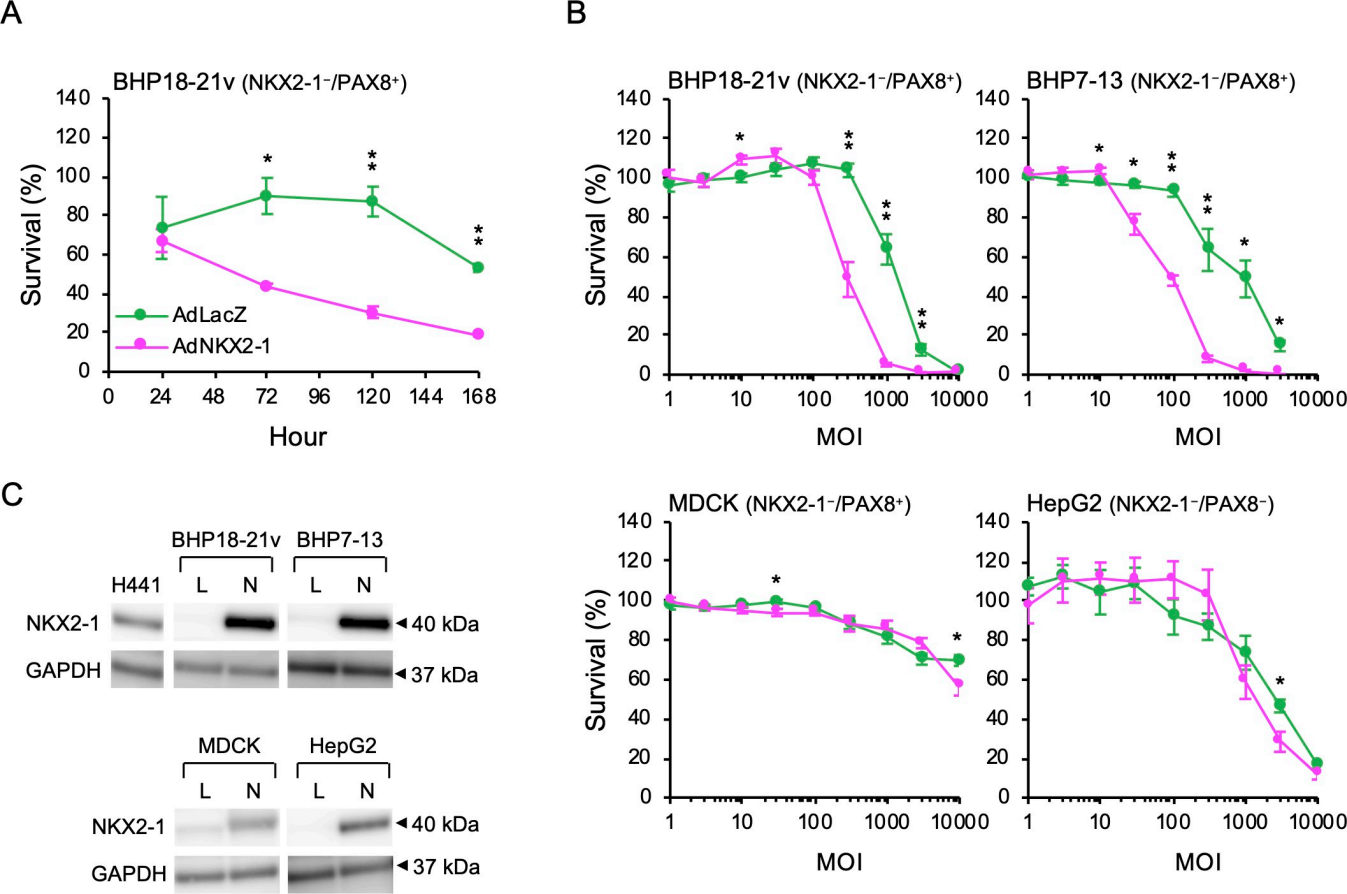
Cell viability after AdNKX2-1 and AdLacZ infection. (A) Time-dependent changes after 1000 MOI adenoviral vectors infection in BHP18-21v cells (n = 9). (B) MOI-dependent changes 120 h after adenoviral vector infection (n = 4–9). The ratios of surviving infected cells to non-infected cells at the same timepoint after infection are shown. Error bars indicate SEM. *p<0.05 and **p<0.01, significant differences between AdNKX2-1 and AdLacZ. (C) Western blots showing NKX2-1 expression 72 h after infection with 300 MOI AdLacZ or AdNKX2-1 (upper panel). Loading control, GAPDH expression (bottom panel). L, AdLacZ; N, AdNKX2-1.

Time-dependent changes in BHP18-21v cell viability after infection with adenoviral vectors were investigated using the CCK-8 assay. Cell viability was measured after 24, 72, 120, and 168 h of incubation with 1000 MOI AdNKX2-1 or AdLacZ. The percentage of surviving infected cells over non-infected cells at a particular time point are shown in Fig 1A. The differences in these percentages between AdNKX2-1- and AdLacZ-infected cells were statistically analyzed. The percentages of surviving AdNKX2-1-infected cells were significantly lower than those of AdLacZ-infected cells 72, 120, and 168 h after infection (*p* = 0.036, = 0.002, and <0.001, respectively).

MOI-dependent changes in AdNKX2-1 or AdLacZ infection-induced cell death were also analyzed (Fig 1B). After incubating with 1, 3, 10, 30, 100, 300, 1000, 3000, or 10000 MOI adenoviral vectors for 120 h, cell viability was measured. The survival percentages of 300, 1000, and 3000 MOI AdNKX2-1-infected BHP18-21v cells were significantly decreased than those of the AdLacZ-infected cells (*p*<0.001, <0.001, and = 0.004, respectively). To investigate whether AdNKX2-1 causes death in another cell line derived from papillary thyroid carcinoma, BHP7-13 cells (NKX2-1^−^/PAX8^+^) were infected with AdNKX2-1 or AdLacZ. The survival percentages of 30, 100, 300, 1000, and 3000 MOI AdNKX2-1-infected BHP7-13 cells were also significantly decreased than those of the AdLacZ-infected cells (*p* = 0.011, <0.001, = 0.002, = 0.016, and = 0.013, respectively). To examine non-thyroid cells, MDCK (NKX2-1^−^/PAX8^+^) and HepG2 cells (NKX2-1^−^/PAX8^−^) were infected with AdNKX2-1 or AdLacZ. In contrast to BHP18-21v and BHP7-13 cells, there were no significant differences between the survival percentage of AdNKX2-1- and AdLacZ-infected cells, except 30 and 1000 MOI infected MDCK cells (*p* = 0.023 and = 0.048, respectively) and 3000 MOI infected HepG2 cells (*p* = 0.017). These results indicated the possibility that AdNKX2-1 infection induces only thyroid cancer cell death.

We analyzed whole cell lysate proteins extracted from 300 MOI AdLacZ- or AdNKX2-1-infected cells using western blotting to confirm the increased NKX2-1 expression due to AdNKX2-1 infection. Fig 1C shows that NKX2-1 was expressed in the positive control, human lung papillary adenocarcinoma (H441) cells, AdNKX2-1-infected BHP18-21v, BHP7-13, MDCK, and HepG2 cells.

### AdNKX2-1-induced apoptosis and necrosis in BHP18-21v cells

**Fig 2:**
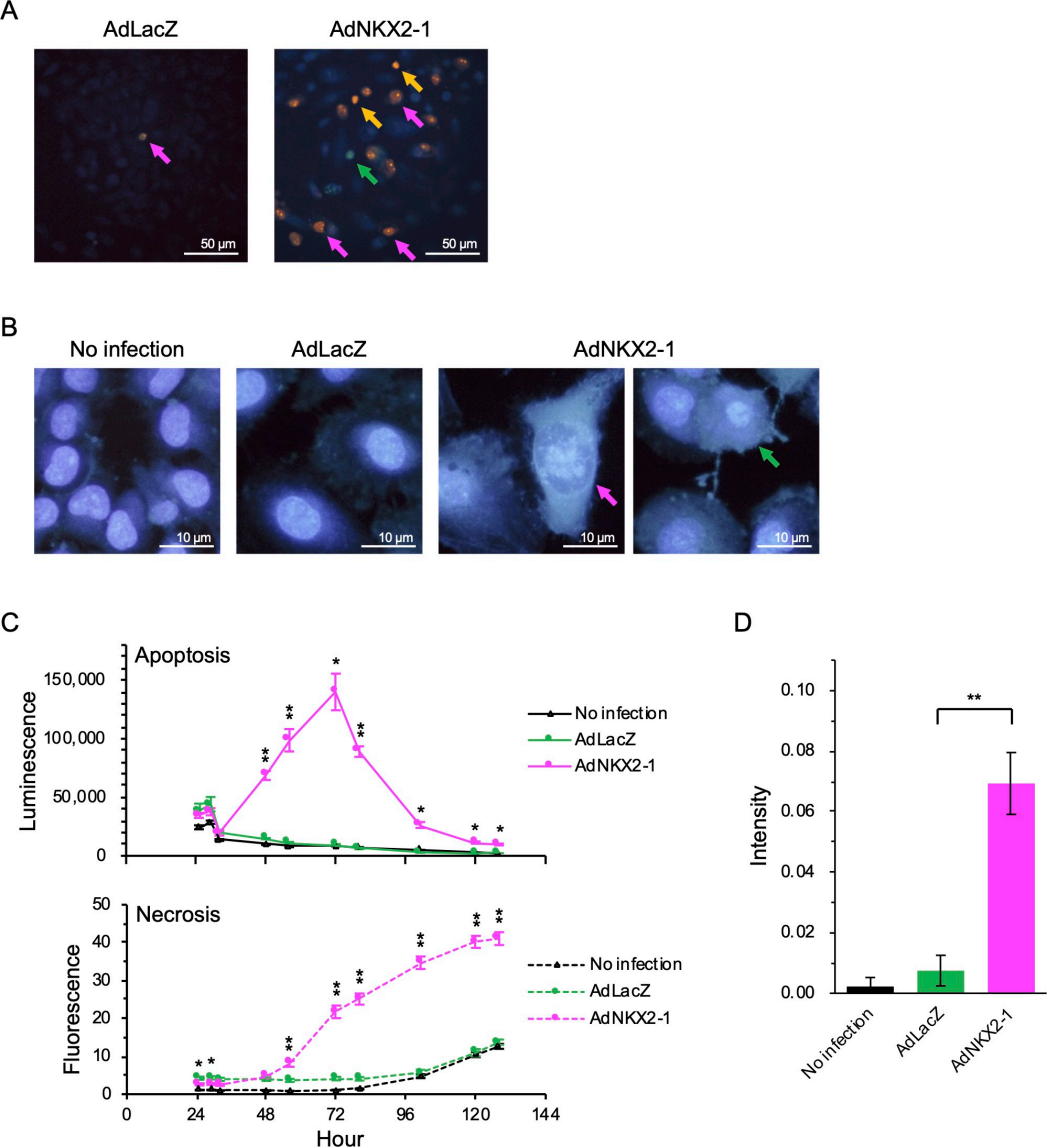
Apoptosis and necrosis detection in BHP18-21v cells after adenoviral vector infection. (A) Acridine orange- and ethidium bromide-stained cells. The green, orange, and magenta arrows represent early apoptotic (acridine orange-stained condensed chromatin), late apoptotic (ethidium bromide-stained condensed chromatin), and necrotic (ethidium bromide-stained normal chromatin) cells, respectively. (B) Hoechst 33342-stained cells. The green and magenta arrows represent an apoptotic cell with a condensed nucleus and a necrotic cell with a ballooning nucleus, respectively. (C) Real-time assay using annexin V (n = 3). The luminescence and fluorescence signals indicate apoptosis and necrosis, respectively. Error bars indicate SEM. *p<0.05 and **p<0.01, significant differences between AdNKX2-1 and AdLacZ. (D) Caspase 3/7 activity-dependent fluorescence intensity 72 h after infection (n = 6). Error bars indicate SEM. **p<0.01, significant differences between AdNKX2-1 and AdLacZ.

To analyze the mechanisms of BHP18-21v cell death induced by AdNKX2-1 infection, acridine orange and ethidium bromide double staining and Hoechst 33342 staining were performed 48 h after 300 MOI adenoviral vector infection to detect the apoptotic and necrotic cells. The acridine orange and ethidium bromide double staining showed only a few necrotic cells among the AdLacZ-infected cells; however, both necrotic and early and late apoptotic cells were observed among the AdNKX2-1-infected cells (Fig 2A). Hoechst 33342 staining revealed apoptotic cells with condensed nuclei and necrotic cells with ballooning nuclei in AdNKX2-1-infected cells, but not in AdLacZ-infected cells (Fig 2B). Real-time assays for detecting apoptosis and necrosis were also performed to analyze time-dependent changes after 300 MOI adenoviral vector infection. The luminescence (apoptosis) and fluorescence (necrosis) signals in the controls (non-infected BHP18-21v cells), AdNKX2-1-infected cells, and AdLacZ-infected cells are shown (Fig 2C). The apoptosis signals in the AdNKX2-1-infected cells were significantly increased than those in AdLacZ-infected cells between 48 and 128 h after infection (*p*≤0.010). On the contrary, the necrosis signals in the AdNKX2-1-infected cells were significantly increased than those in AdLacZ-infected cells 56 h after infection onwards (*p*≤0.007). Caspase activation was assessed to confirm apoptosis in BHP18-21v cells 72 h after adenoviral vector infection. Fig 2D shows caspase 3/7-specific fluorescence intensity in infected and non-infected cells. Caspase activity was significantly higher in 300 MOI AdNKX2-1-infected BHP18-21v cells than that in AdLacZ-infected cells (*p*<0.001). Taken together, these results demonstrate that AdNKX2-1 infection induces both apoptosis and necrosis in BHP18-21v cells.

### GeneChip analysis after AdNKX2-1 infection

**Fig 3:**
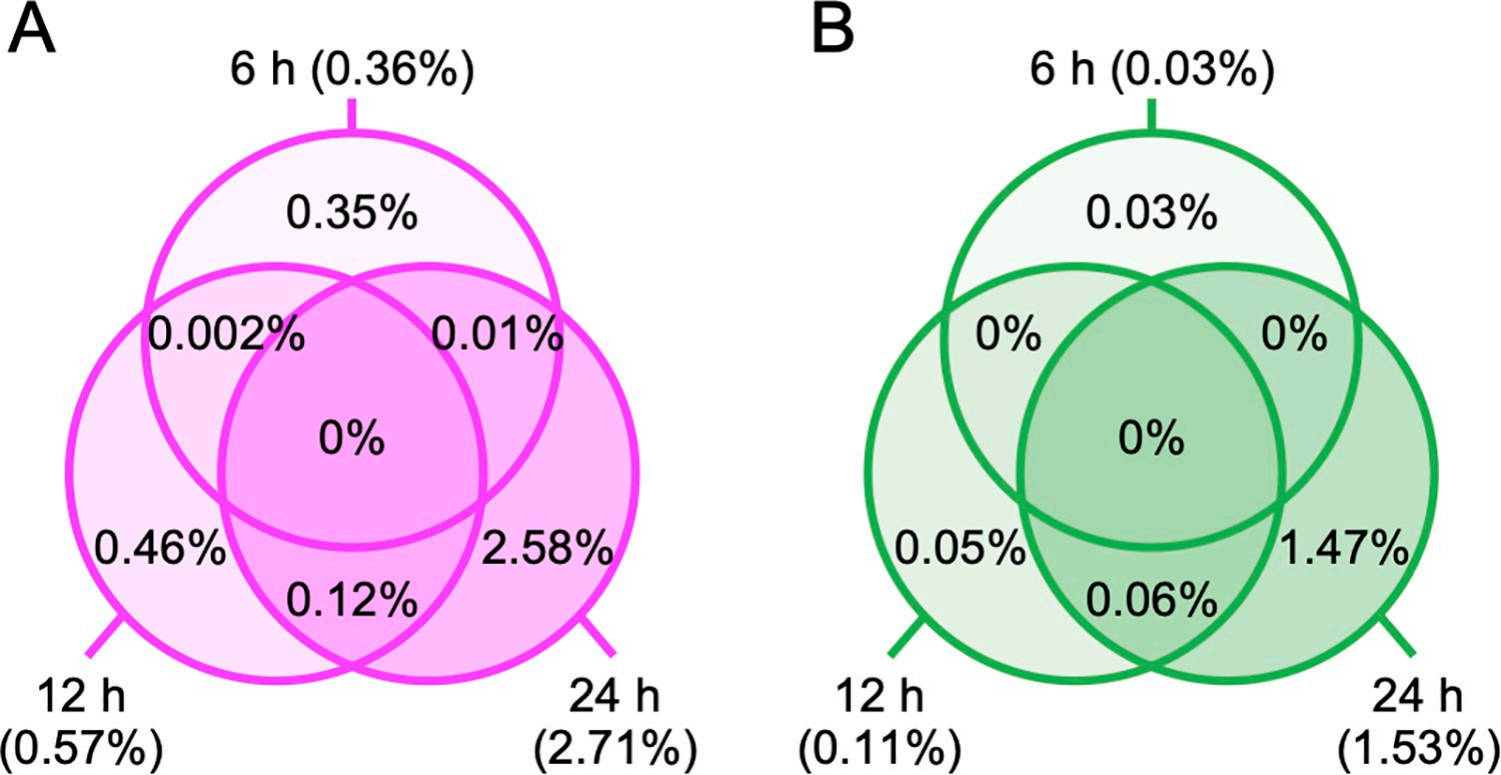
The percentages of genes with largely changed expression after AdNKX2-1 infection. The percentage of genes whose ratio of expression in the AdNKX2-1-infected cells to that in the AdLacZ-infected cells was (A) twice or higher and (B) 0.5-fold or lower in GeneChip analyses 6, 12, and 24 h after 100 MOI adenoviral vector infection. A total of 54,613 transcripts were analyzed. Data with statistically insignificant changes were excluded from the analysis.

To explore the molecules involved in AdNKX2-1 infection-induced BHP18-21v cell death, gene expression after adenoviral vector infection was analyzed comprehensively using GeneChip microarrays. The gene expression in AdNKX2-1-infected cells was compared to that in AdLacZ-infected cells 6, 12, and 24 h after infection. Analyses were performed for 54,613 transcripts. The ratio of gene expression in the AdNKX2-1-infected cells to that in the AdLacZ-infected cells was twice or higher for 199, 314, and 1482 genes 6, 12, and 24 h after infection, respectively (Fig 3A). On the contrary, the ratio was 0.5-fold or less for 15, 62, and 838 genes 6, 12, and 24 h after infection, respectively (Fig 3B). The analysis based on the Gene Ontology resource showed that AdNKX2-1 infection substantially increased the expression of genes categorized into the following categories: regulation of signal transduction, regulation of G-protein coupled receptor protein signaling pathway, negative regulation of cellular process, negative regulation of biological process, telomerase-dependent telomere maintenance, lysine catabolism, and positive regulation of cell proliferation. Table 1 lists the individual genes whose expression was largely increased by AdNKX2-1 infection. The signal intensities of gene expression in AdNKX2-1- and AdLacZ-infected cells and the ratios of the intensities (AdNKX2-1/AdLacZ) at each time point are also shown.

**Table 1:** The list of genes whose expression was largely increased by AdNKX2-1 infection.

Genes	Probe set name	Gene expression								
		AdNKX2-1			AdLacZ			Ratio (AdNKX2-1/AdLacZ)		
		6 h	12 h	24 h	6 h	12 h	24 h	6 h	12 h	24 h
*AKAP28*	228493_at	0.76	1.02	1.65	1.24	0.75	(0.03)	0.61	1.36	47.88
*RGS5*	1555725_a_at	(1.23)	2.65	30.98	(0.77)	(1.15)	0.69	1.58	2.30	45.07
	218353_at	1.24	2.04	21.14	0.76	(0.32)	0.60	1.64	6.46	35.46
	209071_s_at	1.06	3.65	46.39	0.94	0.88	1.32	1.13	4.14	35.26
*ARG2*	203946_s_at	1.12	0.95	17.51	0.88	0.47	0.65	1.27	2.00	26.88
*MMP1*	204475_at	0.93	1.74	22.57	1.07	0.91	1.11	0.88	1.91	20.27
*FLJ10156*	230076_at	1.08	0.76	2.43	0.92	(0.64)	(0.14)	1.18	1.20	16.80
*RRAGC*	242531_at	(0.69)	1.41	1.43	1.31	0.93	(0.09)	0.53	1.51	15.43
*MAP2K5*	211371_at	1.58	3.54	5.09	(0.42)	(0.23)	(0.37)	3.73	15.70	13.74
*cDNA clone IMAGE*:*4816369*	1561353_at	(0.71)	1.57	2.32	(1.29)	1.41	(0.19)	0.55	1.11	12.11
*RGS4*	204337_at	1.04	2.36	4.74	0.96	0.61	0.43	1.08	3.89	11.16
*KRTAP7-1*	1564960_at	0.86	1.72	(1.33)	(1.14)	1.16	(0.12)	0.75	1.48	10.86
*cDNA clone IMAGE*:*2737354*	240177_at	(0.95)	1.95	2.41	(1.05)	1.49	(0.22)	0.90	1.31	10.75
*NRK*	227971_at	(1.02)	2.31	10.58	(0.98)	0.35	1.00	1.05	6.58	10.59

The signal intensities of gene expression in the AdNKX2-1- and AdLacZ-infected cells and the ratios of the intensities (AdNKX2-1/AdLacZ) at each time point are shown. Genes are listed in descending order of ratio 24 h after infection. Numbers in parentheses show signal intensities regarded as insignificant in the statistical investigations using GeneChip microarray. *AKAP28*, 28 kDa A-kinase anchoring protein; *RGS*, regulator of G protein signaling; *ARG2*, arginase 2; *MMP1*, matrix metallopeptidase 1; *FLJ*, full-length long Japan; *RRAGC*, Ras related GTP binding C; *MAP2K5*, mitogen-activated protein kinase kinase 5; *IMAGE*, Integrated Molecular Analysis of Genomes and their Expression; *KRTAP7-1*, keratin associated protein 7–1; *NRK*, Nik related kinase.

For further investigation, we selected three genes, *ARG2*, *RGS4*, and *RGS5* based on the following criteria: there was an apparent expression 6 h after infection, the ratio 12 h after infection was more than twice, and the ratio 24 h after infection was >10-fold. *AKAP28* was excluded even though the ratios were remarkably increased, since the signal intensities in the AdNKX2-1-infected cells did not increase at all. *MMP1* and *MAP2K5* were also excluded because their real-time PCR evaluated expressions did not increase in AdNKX2-1-infected cells. In addition, *NRK* was excluded because its signal intensity was not statistically reproducible in the GeneChip microarray.

### AdNKX2-1-enhanced ARG2, RGS4, and RGS5 expression in BHP18-21v cells

**Fig 4:**
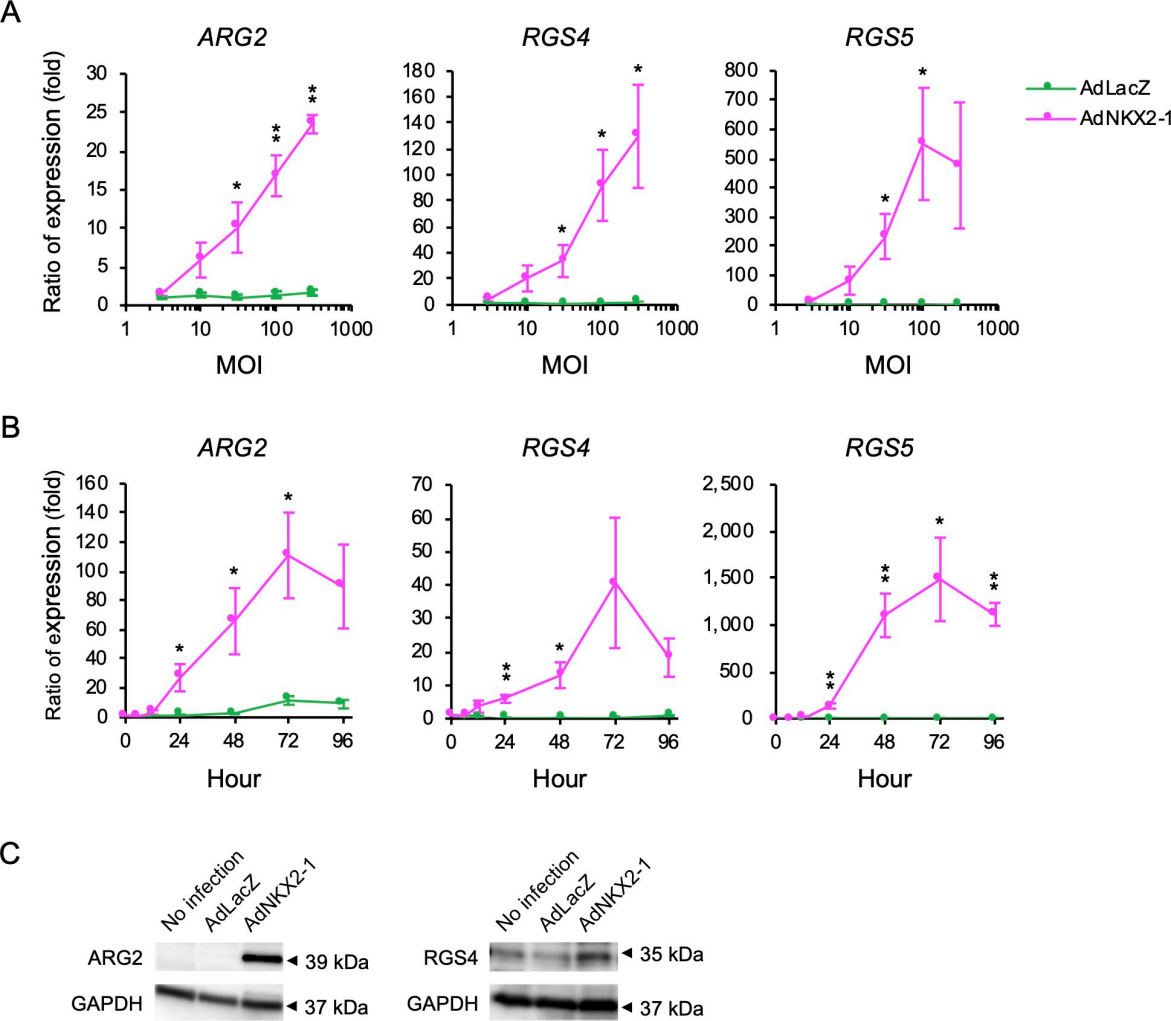
ARG2, RGS4, and RGS5 expression in BHP18-21v cells analyzed using real-time PCR and western blotting. (A) MOI-dependent changes in gene expressions quantified by real-time PCR 72 h after adenoviral vector infection (n = 6). Ratios of gene expressions in infected cells to those in non-infected cells 72 h after infection are shown. (B) Time-dependent changes in gene expressions quantified by real-time PCR after 300 MOI adenoviral vector infection (n = 6). Ratios of gene expressions in infected cells to those in pre-infected cells (0 h) are shown. Error bars indicate SEM. *p<0.05 and **p<0.01, significant differences between AdNKX2-1 and AdLacZ. (C) Western blot showing ARG2 and RGS4 expression 72 h after 300 MOI AdLacZ or AdNKX2-1 infection (upper panel). Loading control, GAPDH expression (bottom panel).

The mRNA levels were quantified using real-time PCR 72 h after infection. The ratios of *ARG2*, *RGS4*, and *RGS5* expression levels in AdNKX2-1- or AdLacZ-infected cells to those in non-infected cells 72 h after infection are shown in Fig 4A. *ARG2*, *RGS4*, and *RGS5* mRNA levels increased in an MOI-dependent manner in AdNKX2-1-infected BHP18-21v cells. *ARG2* (*p* = 0.040, <0.001, and <0.001, respectively) and *RGS4* (*p* = 0.044, = 0.021, and = 0.023, respectively) expression levels after 30, 100, and 300 MOI AdNKX2-1 infection was significantly higher than those after AdLacZ infection. Moreover, *RGS5* expression level in 30 and 100 MOI AdNKX2-1-infected cells were significantly higher than those in AdLacZ-infected cells (*p* = 0.029 and = 0.035, respectively). We also investigated the effect of AdNKX2-1 infection on non-thyroid cells. *ARG2*, *RGS4*, or *RGS5* expressions were not induced 72 h after 30, 100, 300, and 1000 MOI AdNKX2-1 infection in HepG2 cells.

To examine the time course, mRNA levels were quantified 6, 12, 24, 48, 72, and 96 h after 300 MOI adenoviral vector infection. The ratios of *ARG2*, *RGS4*, and *RGS5* expression levels in AdNKX2-1- or AdLacZ-infected cells to those in the pre-infected cells (0 h) are shown in Fig 4B. Time-dependent increases in *ARG2*, *RGS4*, and *RGS5* expression levels were observed up to 72 h in AdNKX2-1-infected cells, but not in AdLacZ-infected cells.

We investigated ARG2, RGS4, and RGS5 protein expression by western blot analysis of whole cell lysates extracted from BHP18-21v cells 72 h after 300 MOI AdLacZ or AdNKX2-1 infection. Fig 4C shows more abundant ARG2 and RGS4 expression in AdNKX2-1- than that in AdLacZ-infected cells. However, the two different antibodies did not show an increase in RGS5 protein expression. These data indicated that ARG2 and RGS4 were increased in BHP18-21v cells by NKX2-1 transduction. Therefore, we selected ARG2 and RGS4 for further investigation using siRNAs.

### Effects of siARG2 and siRGS4 transfection in AdNKX2-1-infected BHP18-21v cells

**Fig 5:**
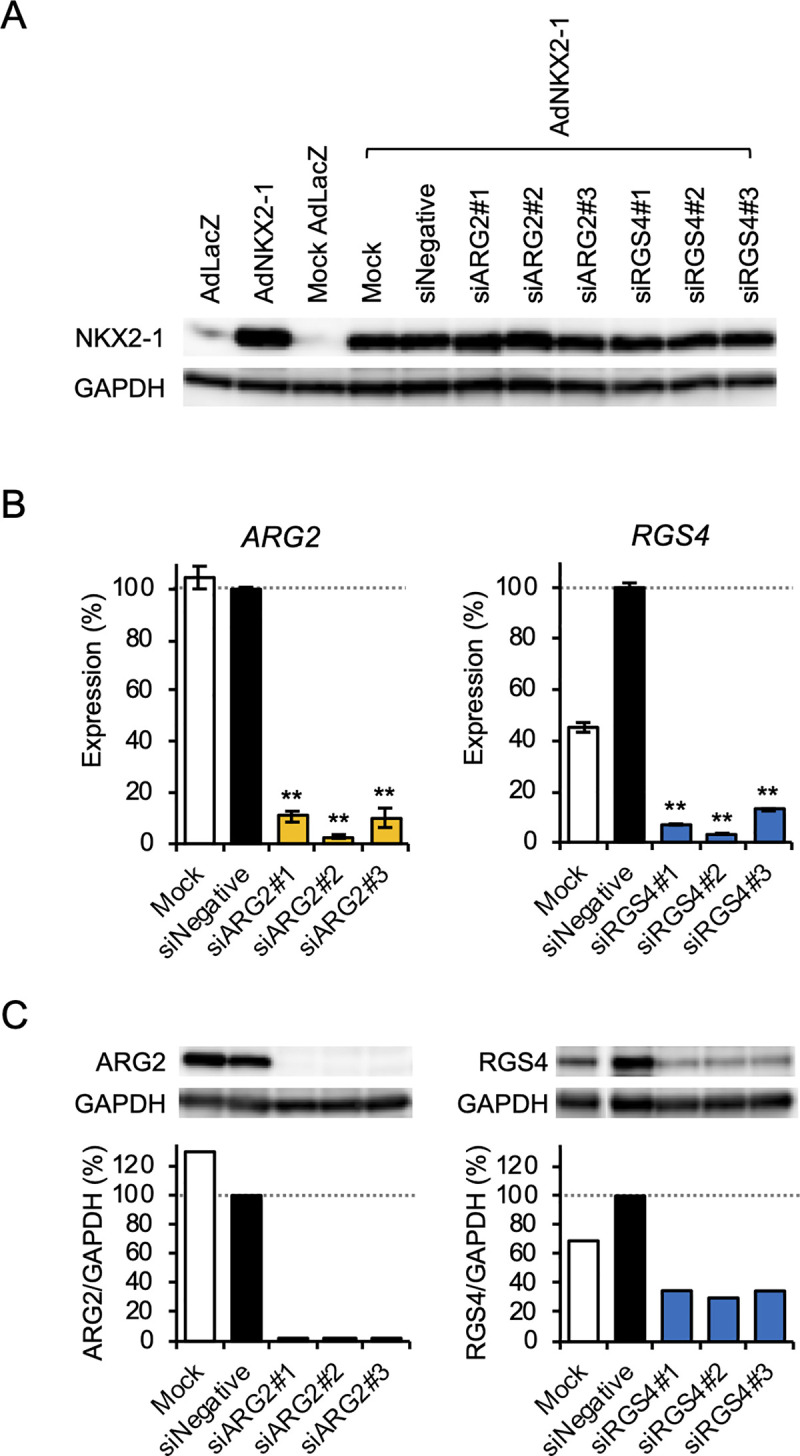
ARG2 and RGS4 expression in siRNA-transfected cells after AdNKX2-1 infection. (A) Western blots showing NKX2-1 and GAPDH expression 72 h after infecting BHP18-21v cells with and without siRNA transfection with 300 MOI AdLacZ or AdNKX2-1. ’Mock’ represents the Opti-MEM^®^ Medium and Lipofectamine^TM^ RNAiMAX exposed, but not siRNA transfected cells. (B) *ARG2* and *RGS4* mRNA expressions in siARG2- and siRGS4-transfected cells, respectively, are shown in percent of control (n = 4). siNegative-transfected cells were used as the control. All the siRNAs, #1, #2, and #3, targeting *ARG2* and *RGS4* efficiently downregulated each of the gene expressions. Error bars indicate SEM. **p<0.01, significant differences between siNegative and siARG2 or siRGS4. (C) Western blot showing ARG2 and RGS4 expression 72 h after 300 MOI AdNKX2-1 infection. The ratios (%) of protein expression in the siRGS4#1–#3-transfected cells compared with that in siNegative-transfected cells are shown.

To demonstrate the efficiency of siRNA-mediated gene silencing, BHP18-21v cells were transfected with siRNAs targeting three different mRNA regions in each gene: siARG2#1−#3 and siRGS4#1−#3. Non-specific siRNA was used as a negative control (siNegative). Transfection with these siRNAs did not affect NKX2-1 expression level in BHP18-21v cells (Fig 5A). The mRNA expression was quantified using real-time PCR after 100 MOI AdNKX2-1 infection. Both siRNAs targeting *ARG2* and *RGS4* efficiently downregulated mRNA expression in AdNKX2-1-infected BHP18-21v cells (Fig 5B). The western blot findings also confirmed ARG2 and RGS4 expression suppression in siRNA-transfected BHP18-21v cells after AdNKX2-1 infection (Fig 5C). The ratios (%) of protein expression in the cells transfected with siARG2#1–#3 or siRGS4#1–#3 compared with that in cells transfected with siNegative were 1.5–1.9% or 30.1–35.1%, respectively.

**Fig 6:**
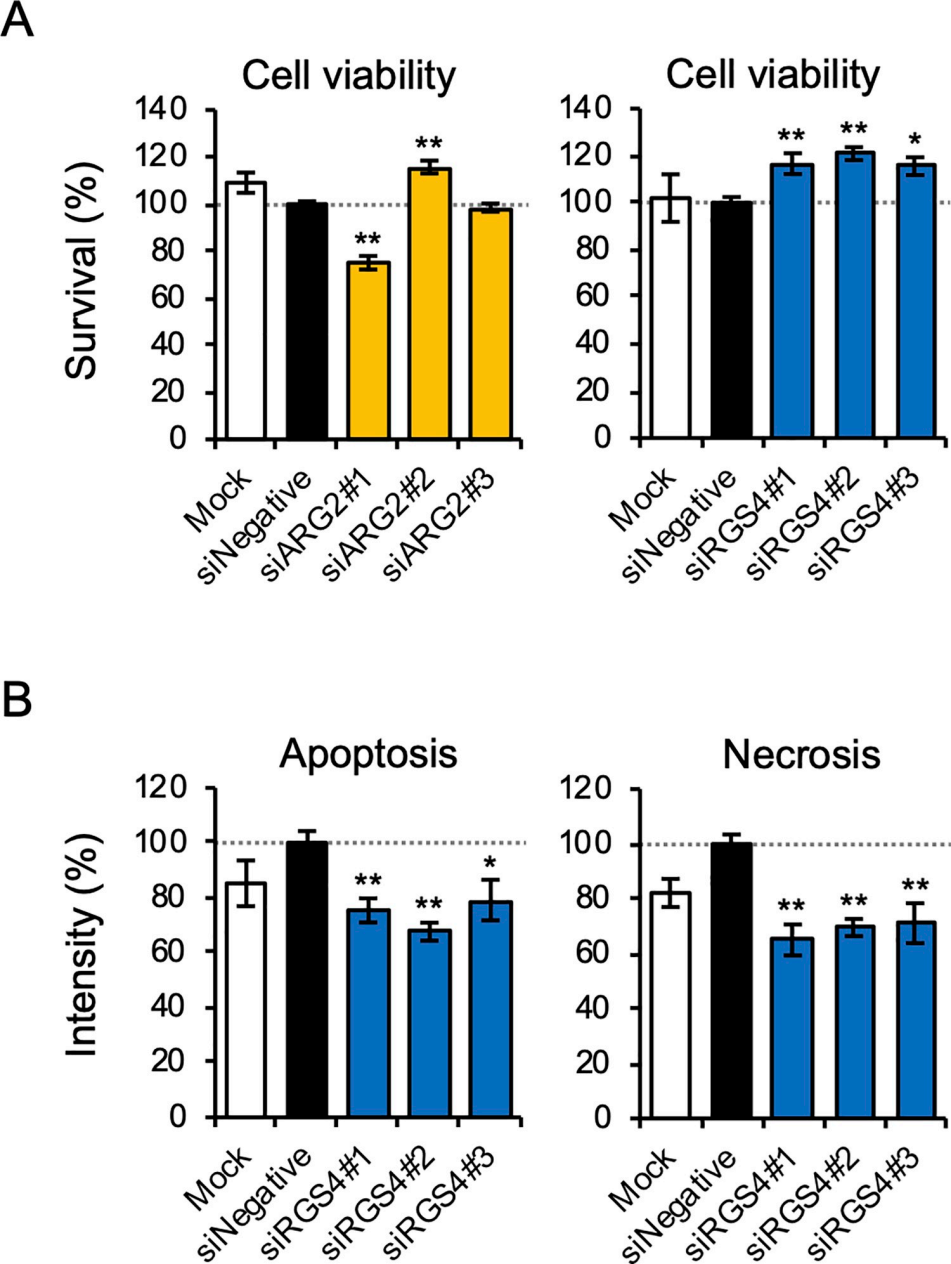
The effects of siARG2 or siRGS4 on AdNKX2-1-induced cell death, apoptosis, and necrosis. The number of surviving cells and apoptosis and necrosis signal intensities in siRNA-transfected cells are shown as a percentage of the control. siNegative-transfected cells were used as controls. ’Mock’ represents the Opti-MEM^®^ Medium and Lipofectamine^TM^ RNAiMAX exposed, but not siRNA transfected cells. Error bars indicate SEM. *p<0.05, **p<0.01, significant differences between siNegative and siARG2 or siRGS4. (A) Viability of siARG2- or siRGS4-transfected cells 72 h after 300 MOI AdNKX2-1 infection (n = 9). (B) Apoptosis and necrosis in siRGS4-transfected cells 72 and 96 h, respectively, after 300 MOI AdNKX2-1 infection (n = 9).

To investigate the effects of ARG2 and RGS4 on cell death due to AdNKX2-1 infection, siRNA-transfected BHP18-21v cell viability was measured 72 h after 300 MOI AdNKX2-1 infection. The percentages of the ratios of surviving cells in the siARG2- or siRGS4-transfected cells to those in the negative control (siNegative-transfected cells) are shown in Fig 6A. The viability of siARG2#2-, siARG2#1-, and siARG2#3-transfected cells was significantly higher (115.7±2.7%, *p*<0.001), lower (75.1±2.9%, *p*<0.001), and similar (98.5±1.7%, *p* = 0.932) compared with that of the negative control. In contrast, the viability of siRGS4#1-, siRGS4#2-, and siRGS4#3-transfected cells was significantly higher (116.6±4.4%, 120.9±2.8%, and 115.6±3.8%; *p* = 0.007, = 0.001, and = 0.012; respectively) than that of the negative control. These results indicated that suppressing RGS4 expression significantly inhibits AdNKX2-1 infection-induced cell death, although the three siARG2s did not consistently affect cell death.

To examine the effects of RGS4 on AdNKX2-1 infection-induced apoptosis and necrosis, apoptosis and necrosis were analyzed using real-time assays in siRGS4-transfected BHP18-21v cells 72 and 96 h, respectively, after 300 MOI AdNKX2-1 infection. These time points were selected based on the results of the preceding real-time assays without siRNA transfection (Fig 2C). The ratios (%) of luminescence (apoptosis) and fluorescence (necrosis) intensity in the siRGS4-transfected cells significantly decreased (67.6±3.3% [*p*<0.001] to 79.1±7.4% [*p* = 0.018] and 65.1±5.6% [*p*<0.001] to 71.2±7.3% [*p* = 0.001], respectively) compared with those in the negative control (Fig 6B). These results indicated that RGS4 expression suppression significantly inhibited AdNKX2-1 infection-induced apoptosis and necrosis.

## Discussion

In general, thyroid cancer has a good prognosis. However, poorly differentiated thyroid carcinoma (PDTC), which constitutes 2–15% of all thyroid cancers, induces distant metastases in up to 85% cases [16]. Moreover, the one-year disease-specific survival rate of anaplastic thyroid carcinoma, one of the most virulent and aggressive malignancies, is 34.3% [17] because of the rapid invasion of adjacent structures and metastasis throughout the body. Furthermore, NKX2-1 expression decreases in dedifferentiated thyroid carcinoma cells [8]. This study demonstrated that *NKX2-1* transduction into dedifferentiated thyroid carcinoma cells that do not express NKX2-1 using an adenoviral vector induces cell death. We also showed that NKX2-1 re-expression caused apoptosis and necrosis in these cells.

In this study, BHP18-21v and BHP7-13 cell death was observed after AdNKX2-1 infection. As these thyroid cancer cells expressed PAX8 but not NKX2-1, cell death was seemingly due to the effects induced by NKX2-1 re-expression. The analyses of non-thyroid cell viabilities, however, showed that AdNKX2-1 infection did not cause HepG2 liver cell death, which does not express PAX8 or NKX2-1. In addition, cell death was not induced in MDCK kidney cells expressing PAX8, but not NKX2-1, whose expression pattern is identical to that of the thyroid cancer cells used in this study. Thus, non-thyroid cell death was not observed in this study, despite PAX8 expression. These findings indicated that AdNKX2-1-induced cell death was an exclusive effect on thyroid cancer cells, although we examined only two papillary thyroid carcinoma derived cell lines. We have previously reported that AdNKX2-1 infection induced TG promoter activation in thyroid cancer cells not expressing NKX2-1, but not in MDCK cells [13]. Although both NKX2-1 and PAX8 play key roles in activating the TG promoter, co-expression of these two genes did not activate this promoter in non-thyroid cells. The results of our previous study implied that the difference in TG promoter activation among tissues was related to epigenomic distinction. Similar mechanisms may account for thyroid cancer cell death induction in this study.

There are three distinct cell death morphologies: apoptosis, necrosis, and autophagy-associated cell death [18]. Apoptosis and necrosis assays using four different methods demonstrated that AdNKX2-1 infection causes both apoptosis and necrosis in BHP18-21v cells. Griesing *et al*. have reported that NKX2-1-regulated microRNA induces apoptosis in lung adenocarcinoma cells [19]. In addition, *NKX2-1* transduction increases cellular apoptosis in non-small cell lung cancer, suggesting that it might serve as a tumor suppressor gene [20]. Moreover, the necrosis signal increased after the increased apoptosis signal in real-time assays. Slow cell death, defined as necrosis-like death or delayed necrosis, occurs when caspases are inhibited or absent, and thus occurs later than the approximate time of apoptosis [21]. Necroptosis is defined as regulated necrosis dependent on receptor-interacting protein kinase (RIPK) 1 and/or RIPK3 activity [22]. Vanden Berghe *et al*. have described regulated necrosis as a genetically controlled cell death process eventually resulting in cellular leakage [18]. It is triggered by death ligands such as tumor necrosis factor (TNF), Fas ligand (FasL), and TNF-related apoptosis inducing ligand (TRAIL), particularly when apoptosis is inhibited. Nehs *et al*. have previously reported necroptosis related to radiation-induced cell death in anaplastic thyroid cancer [23]. In this study, necrosis was detected in the process of programmed cell death caused by AdNKX2-1, implying that necrosis in our study was necroptosis. Necroptosis is incompatible with apoptosis because it is identified as a backup and alternative to apoptosis [18]. However, Lin *et al*. have recently reported the simultaneous apoptosis and necroptosis induction by herbal medicines in HepG2 cells [24].

*NKX2-1* is a homeobox gene that encodes a family of homeodomain-containing transcription factors that play important roles in the early embryo, including cell and tissue identity establishment and cell proliferation, differentiation, and survival regulation [25]. Various functions of homeobox genes, such as prospero homeobox 1 (*PROX1)* [26], zinc finger E-box binding homeobox 1 (*ZEB1*) [27], and NK-like (NKL) homeobox genes [28], have been reported. Homeodomain-containing proteins can both activate and repress gene transcription in a context-dependent manner [26]. In this study, comprehensive gene analysis using GeneChip microarrays after AdNKX2-1 infection identified genes with greatly increased expression. These genes belong to several categories. These results indicate that homeobox gene expression diversely regulates the expression levels of multiple genes in thyroid cancer.

Among the individual genes increased in response to AdNKX2-1, we focused on *ARG2*, *RGS4*, and *RGS5*. *ARG2* is an *NKX2-1*-related gene [29], and *RGS4* and *RGS5* are NKX2-1 targets [30]. ARG2, an arginase isoform, converts arginine to ornithine and urea, indicating that increased arginase expression induces arginine reduction, which plays a pivotal role in cellular physiology as it is involved in numerous cellular metabolic and signaling pathways. Thus, arginine deprivation is a potential anti-cancer therapy [31]. A relationship has been established between ARG2 expression and cancer in various organs, including the thyroid [32–34]. However, these studies did not show a universal effect of ARG2 on tumor proliferation or progression. Apoptosis or non-apoptotic cell death has been reported in not only arginase-mediated arginine deprivation therapy [35–37], but also arginase inhibition [32, 33, 38]. Whether arginase overexpression inhibits or induces cancer progression might depend on the context, cell characteristics, tissue types, and cancer progression stages. In this study, the conflicting effects of ARG2 on cell death, determined using three *ARG2*-targeting siRNAs, implied that ARG2 has two functions. In addition, NKX2-1-induced increased ARG2 expression might play a role other than affecting cell death.

RGS proteins modulate the physiological actions of many neurotransmitters, hormones, and other signaling molecules. Human RGS comprises a family of 20 canonical proteins that bind directly to G protein-coupled receptors or G protein complexes to limit the span of their signaling events, which regulate all aspects of cell and organ physiology [39]. RGS4, known to have multiple isoforms, is selectively expressed in the heart and brain and responds to cardiovascular stress and modulates neuronal signaling [39, 40]. RGS4 expression suppresses breast cancer migration, invasion, and proliferation [41, 42], and *RGS4* reduces the risk of bladder cancer with an increasing number of variant alleles [43]. We found that suppressing RGS4 expression by siRNA transfection reduced cell death, apoptosis, and necrosis induced by restored NKX2-1 expression. The role of RGS4 in cancer apoptosis remains controversial. Xue *et al*. have reported that RGS4 reduces the proliferation, migration, and invasion of melanoma cells and apoptosis rates are decreased in a low RGS4 environment [44]. Xiao *et al*. have found that microRNA-107 overexpression in hepatocellular carcinoma cells suppresses cellular proliferation, invasion, migration, and colony-forming ability, but promotes apoptosis and G1 phase arrest by modulating RGS4 expression [45]. These findings indicate that RGS4 overexpression induces apoptosis in cancer cells. In contrast, He *et al*. have found increased RGS4 protein levels in non-small cell lung cancer cells [46]. They showed that RGS4 knockdown inhibits cell proliferation and induces apoptosis, and that RGS4 negatively regulates the tumor suppressor, microRNA-16. *RGS4* overexpression inhibits microRNA-3663-3p expression in thyroid cancer and increases RPL34-AS1 expression, a long non-coding RNA, which induces apoptosis and inhibits proliferation and invasion in papillary thyroid cancer cells [47]. These findings indicate that RGS4 functions vary among cancer types. Our results indicate that RGS4 partially suppresses the survival of dedifferentiated thyroid cancer cells. Since the expression of many genes, in addition to *RGS4*, was altered by NKX2-1 overexpression, it would affect cell death.

RGS5 is found in many tissues, where it controls vascular remodeling [39] and is responsible for abnormal tumor vascular morphology [48]. Altman *et al*. have found that RGS5 expression reduces ovarian cancer cell proliferation, and that more mice bearing RGS5-expressing tumors survive compared with controls [49]. They suggested that these tumor-suppressive effects are independent of the role of RGS5 in vascular normalization and remodeling. Xu *et al*. have also showed that RGS5 inhibits human lung cancer cells [50]. These findings suggest a relationship between RGS5 and cancer, although we could not find an effect of increased *RGS5* expression on BHP18-21v cells.

We demonstrated that *NKX2-1* expression using adenoviral vectors led to *NKX2-1*-non-expressing thyroid carcinoma cell death. Abate-Shen has reported that both loss and gain of homeobox gene expression are associated with tumorigenesis and most reported cases of deregulated homeobox gene expression in cancer conform to the rule that the homeobox genes normally expressed in undifferentiated cells are upregulated in cancer, while those normally expressed in differentiated tissues are downregulated [51]. *NKX2-1* has both pro- and anti-oncogenic activities in lung cancer [52]. Yamaguchi *et al*. have showed that *NKX2-1* acts as a lineage-survival oncogene and cancer progression suppressor in lung adenocarcinomas [12]. NKX2-1 functions as a double-edged sword in lung adenocarcinoma pathogenesis as it is transcribed under the influence of various transcription factors, and its transcription regulatory activities are modulated in a context-dependent manner by co-operating transcription factors as well as protein modifications. Li *et al*. have reported that the developmental transcription factors forkhead box A2 (FOXA2) and caudal type homeobox 2 (CDX2) function cooperatively with NKX2-1 as important regulators in inhibiting lung adenocarcinoma metastasis [53]. They described these three transcription factors to be consistently downregulated in metastatic cells than in non-metastatic cells. Tomida *et al*. have reported that enforced distal-less homeobox 4 (*DLX4*) gene expression, which is reduced in a highly metastatic lung cancer cell line and mostly associated with favorable prognosis in lung cancer patients, reduces lung cancer cell motility and invasion in vitro as well as their metastasis in vivo through both hematogenous and lymphogenous routes [54]. We have previously reported that NKX2-1 re-expression in dedifferentiated thyroid cancer cells is induced by histone deacetylase (HDAC) inhibitors [55]. Gene expression is regulated by histone acetyltransferase (HAT) and HDAC. HAT is deregulated or mutated in several cancers [56]. Whereas histone acetylation opens the chromatin structure, allowing for the binding of transcription factors, and leads to gene expression, histone deacetylation confers a close chromatin structure and inhibits gene transcription. Thus, HDAC inhibitors maintain an open chromatin conformation and induce transcriptional activation. HDAC inhibitors also promote caspase-mediated apoptosis in thyroid cancer cells [57]. Accordingly, exploring factors that effectively induce *NKX2-1* re-expression would lead to novel approaches for treating dedifferentiated thyroid cancer.

In conclusion, forced NKX2-1 expression using adenoviral vectors induced cell death in dedifferentiated thyroid cancer cells not expressing NKX2-1. In addition, NKX2-1 expression induction caused apoptosis and necrosis in these cells. RGS4 expression suppression partially inhibited cell death, apoptosis, and necrosis after NKX2-1 re-expression in dedifferentiated thyroid carcinoma cells. Our results suggest that RGS4 plays a partial role in suppressing tumor progression. These findings could provide new perspectives on progression mechanisms and novel therapeutic approaches for dedifferentiated thyroid carcinoma.

One limitation of the present study is that we examined only two papillary thyroid carcinoma derived cell lines and were unable to assess the effects of NKX2-1 overexpression in other thyroid cancer cells, such as PDTC or anaplastic thyroid carcinoma.
